# Use of an efficient unbiased estimator for finite population mean

**DOI:** 10.1371/journal.pone.0270277

**Published:** 2022-07-01

**Authors:** Javid Shabbir, Ronald Onyango

**Affiliations:** 1 Department of Statistics, Quaid-i-Azam University, Islamabad, Pakistan; 2 Department of Applied Statistics, Financial Mathematics and Actuarial Science, Jaramogi Oginga Odinga University of Science and Technology, Bondo, Kenya; Government College University Faisalabad, PAKISTAN

## Abstract

In this study, we propose an improved unbiased estimator in estimating the finite population mean using a single auxiliary variable and rank of the auxiliary variable by adopting the Hartley-Ross procedure when some parameters of the auxiliary variable are known. Expressions for the bias and mean square error or variance of the estimators are obtained up to the first order of approximation. Four real data sets are used to observe the performances of the estimators and to support the theoretical findings. It turns out that the proposed unbiased estimator outperforms as compared to all other considered estimators. It is also observed that using conventional measures have significant contributions in achieving the efficiency of the estimators.

## 1. Introduction

In literature, many researchers have constructed or modified several forms of ratio, product, and regression type estimators by using the auxiliary information in estimating the finite population mean. The auxiliary information can be used either at survey stage or designing stage or estimation stage or at all stages to enhance the precision of the estimators by taking the advantage of correlation between the study variable and the auxiliary variable. In this study, we use the auxiliary variable as well as rank of the auxiliary variable at estimation stage to estimate the finite population mean. [1] were the pioneer whom used the idea of ratio of the study variable and the auxiliary variable in estimating the population mean. Singh and Singh [2] suggested the [1] type estimator when some parameters of the auxiliary variable are known in advance. [3] slightly modified the idea of [1] and suggested a new estimator for estimating the population mean. [4] used the known population parameters of the auxiliary variable in their suggested estimator for mean estimation. [5] extended the [1] estimator by using two auxiliary variables to estimate the population mean. [6, 7] modified the [1] type estimator for mean estimation in simple and stratified sampling. [8] have given justification in their proposed estimator by using dual use of the auxiliary variable in their study. [9] used the dual auxiliary variable in estimating the mean of the sensitive variable under randomized response technique (RRT). [10] modified the existing ratio estimator by using the dual auxiliary information for mean estimation. [11] suggested a difference type exponential estimator based on dual auxiliary variable for mean estimation. Recently [12] suggested a difference type estimator using the dual auxiliary variable under non-response in simple random sampling.

There are several estimators exist in literature which give the biased results and consequently variance or MSE tend to be inflated. This serious drawback encouraged us to construct the unbiased estimator which should be better than other considered estimators in literature. So combining the ideas of [1] and [2], we suggest an improved unbiased estimator for estimating the finite population mean.

In Section 2, we introduce some useful notations and symbols. Section 3 gives the existing estimators in literature. The proposed estimator is discussed in Section 4. The numerical results based on four real data sets are mentioned in Section 5. The conclusion is given in Section 6.

## 2. Symbols and notations

Consider a finite population Λ = Λ{Λ_1_, Λ_2_, …, Λ_*N*_} of *N* units. A simple random sample without replacement (SRSWOR) is used to draw a sample of size *n* units from a population. Let *y*_*i*_, *x*_*i*_, and *r*_*i*_ be the observed values of the study variable (*Y*), the auxiliary variable (*X*) and rank of the auxiliary variable (*R*) respectively. Let $\bar{y}=\sum_{i=1}^{n}y_{i}/n$, $\bar{x}=\sum_{i=1}^{n}x_{i}/n$ and $\bar{r}=\sum_{i=1}^{n}r_{i}/n$ respectively be the sample means corresponding to the population means $\bar{Y}=\sum_{i=1}^{N}y_{i}/N$, $\bar{X}=\sum_{i=1}^{N}x_{i}/N$ and $\bar{R}=\sum_{i=1}^{N}r_{i}/N$. Let $s_{y}^{2}=\sum_{i=1}^{n}{\left(y_{i}-\bar{y}\right)}^{2}/(n-1)$, $s_{x}^{2}=\sum_{i=1}^{n}{\left(x_{i}-\bar{x}\right)}^{2}/(n-1)$, and $s_{r}^{2}=\sum_{i=1}^{N}{\left(r_{i}-\bar{r}\right)}^{2}/(n-1)$ respectively be the sample variances corresponding to population variances $S_{y}^{2}=\sum_{i=1}^{N}{\left(y_{i}-\bar{Y}\right)}^{2}/(N-1)$, $S_{x}^{2}=\sum_{i=1}^{N}{\left(x_{i}-\bar{X}\right)}^{2}/(N-1)$, and $S_{r}^{2}=\sum_{i=1}^{N}{\left(r_{i}-\bar{R}\right)}^{2}/(N-1)$. Let $C_{y}=S_{y}/\bar{Y}$, $C_{x}=S_{x}/\bar{X}$, and $C_{r}=S_{r}/\bar{R}$ be the coefficients of variation of *Y*, *X*, and *R* respectively. Let *ρ*_*yx*_ = *S*_*yx*_ / (*S*_*y*_ *S*_*x*_), *ρ*_*yr*_ = *S*_*yr*_ / (*S*_*y*_ *S*_*r*_), and *ρ*_*xr*_ = *S*_*xr*_ / (*S*_*x*_ *S*_*r*_), be the correlation coefficients between their respective subscripts, where $S_{yx}=\sum_{i=1}^{N}\left(y_{i}-\bar{Y}\right)\left(x_{i}-\bar{X}\right)/(N-1)$, $S_{yr}=\sum_{i=1}^{N}\left(y_{i}-\bar{Y}\right)\left(r_{i}-\bar{R}\right)/(N-1)$, and $S_{xr}=\sum_{i=1}^{N}\left(x_{i}-\bar{X}\right)\left(r_{i}-\bar{R}\right)/(N-1)$ be the population covariances between their respective subscripts. Corresponding sample covariances are $s_{yx}=\sum_{i=1}^{n}\left(y_{i}-\bar{y}\right)\left(x_{i}-\bar{x}\right)/(n-1)$, $s_{yr}=\sum_{i=1}^{n}\left(y_{i}-\bar{y}\right)\left(r_{i}-\bar{r}\right)/(n-1)$, and $s_{xr}=\sum_{i=1}^{n}\left(x_{i}-\bar{x}\right)\left(r_{i}-\bar{r}\right)/(n-1)$.

We define the following relative error terms to derive bias and MSE or variance expressions.

Let $\Psi_{0}=\frac{\bar{y}}{\bar{Y}}-1$ and $\Psi_{1}=\frac{\bar{x}}{\bar{X}}-1$, $\Psi_{2}=\frac{\bar{r}}{\bar{R}}-1$, $\Psi_{3}=\frac{s_{yx}}{S_{yx}}-1$, such that *E*(Ψ_*i*_) = 0, (*i* = 0,1,2,3), $E(\Psi_{0}^{2})=ϒC_{y}^{2}$, $E(\Psi_{1}^{2})=ϒC_{x}^{2}$, $E(\Psi_{2}^{2})=ϒC_{r}^{2}$, $E(\Psi_{3}^{2})=ϒ\left(\frac{\Delta_{220}}{\rho_{yx}}-1\right)$, *E*(Ψ_0_Ψ_1_) = Υ*C*_*yx*_, *E*(Ψ_0_Ψ_2_) = Υ*C*_*yr*_, $E(\Psi_{0}\Psi_{3})=ϒC_{y}\frac{\Delta_{210}}{\rho_{yx}}$, *E*(Ψ_1_Ψ_2_) = Υ*C*_*xr*_, $E(\Psi_{1}\Psi_{3})=ϒC_{yx}\frac{\Delta_{120}}{\rho_{yx}}$, $E(\Psi_{2}\Psi_{3})=ϒC_{r}\frac{\Delta_{102}}{\rho_{yr}}$. where *C*_*yx*_ = *ρ*_*yx*_*C*_*y*_*C*_*x*_, *C*_*yr*_ = *ρ*_*yr*_*C*_*y*_*C*_*r*_, *C*_*xr*_ = *ρ*_*xr*_*C*_*x*_*C*_*r*_, $\Delta_{abc}=\frac{\mu_{abc}}{\mu_{200}^{a/2}\mu_{020}^{b/2}\mu_{002}^{c/2}}$, $\mu_{abc}=\frac{1}{\left(N-1\right)}\sum_{i=1}^{N}{\left(y_{i}-\bar{Y}\right)}^{a}{\left(x_{i}-\bar{X}\right)}^{b}{\left(r_{i}-\bar{R}\right)}^{c}$, and $ϒ=\left(\frac{1}{n}-\frac{1}{N}\right)$.

## 3. Existing estimators

Now we discuss some well-known estimators in estimating the finite population mean.

1. The usual sample mean estimator is ${\bar{y}}_{\left(0\right)}=\bar{y}$, and its variance, is given by
$$Var({\bar{y}}_{\left(0\right)})=ϒ{\bar{Y}}^{2}C_{y}^{2}.$$ (1)
2. A general class of Hartley-Ross unbiased type estimators, is given by
$${\bar{y}}_{\left(G\right)}^{\left(U\right)}={\bar{k}}^{\left(j\right)}{\bar{X}}^{\left(j\right)}+\frac{n(N-1)}{N(n-1)}\left(\bar{y}-{\bar{k}}^{\left(j\right)}{\bar{x}}^{\left(j\right)}\right),$$ (2)
  where ${\bar{x}}^{\left(j\right)}=c_{}\bar{x}+d$, ${\bar{X}}^{\left(j\right)}=c_{}\bar{X}+d$, ${\bar{k}}^{\left(j\right)}=\frac{1}{n}\sum_{i=1}^{n}k_{i}^{\left(j\right)}$, $k_{i}^{\left(j\right)}=\frac{y_{i}}{x_{i}^{\left(j\right)}}$, $x_{i}^{\left(j\right)}=c_{}x_{i}+d$, $E(y_{i})=\bar{Y}$, $E(k_{i}^{\left(j\right)})={\bar{K}}^{\left(j\right)}$, ${\bar{K}}^{\left(j\right)}=\frac{1}{N}\sum_{i=1}^{N}k_{i}^{\left(j\right)};$ *j* = 0, 1, 2; *c* and *d* are the known population parameters of the auxiliary variable which may be coefficient of variation (*C*_*x*_), coefficient of skewness (*β*_1*x*_), coefficient of kurtosis (*β*_2*x*_) and correlation coefficient (*ρ*_*yx*_).

Using the assumption $\frac{n(N-1)}{N(n-1)}\approx 1$ and $\left({\bar{Y}}^{\left(j\right)}-{\bar{K}}^{\left(j\right)}{\bar{X}}^{\left(j\right)}\right)\approx 0$, an unbiased general estimator is given by

$${\bar{y}}_{\left(G\right)}^{\left(U\right)}\approx \bar{y}+{\bar{k}}^{\left(j\right)}\left({\bar{X}}^{\left(j\right)}-{\bar{x}}^{\left(j\right)}\right).$$ (3)

The variance of ${\bar{y}}_{\left(G\right)}^{\left(U\right)}$, is given by

$$Var({\bar{y}}_{\left(G\right)}^{\left(U\right)})\approx ϒ\left[S_{y}^{2}+{\bar{K}}^{(j)2}S_{x^{\left(j\right)}}^{2}-2{\bar{K}}^{\left(j\right)}S_{yx^{\left(j\right)}}\right],$$ (4)

where $S_{x^{\left(j\right)}}^{2}=\frac{1}{\left(N-1\right)}\sum_{i=1}^{N}{\left(x_{i}^{\left(j\right)}-{\bar{X}}^{\left(j\right)}\right)}^{2}$, and $S_{yx^{\left(j\right)}}=\frac{1}{\left(N-1\right)}\sum_{i=1}^{N}\left(y_{i}-\bar{Y})(x_{i}^{\left(j\right)}-{\bar{X}}^{\left(j\right)}\right)=\rho_{yx^{\left(j\right)}}S_{y}S_{x^{\left(j\right)}}$.

Note:

1. Put *c* = 1, *d* = 0, in (3), so *j* = 0, i.e. ${\bar{x}}^{\left(0\right)}=\bar{x}{,}_{}{\bar{X}}^{\left(0\right)}=\bar{X}$, ${\bar{k}}^{\left(0\right)}=\frac{1}{n}\sum_{i=1}^{n}k_{i}^{\left(0\right)},$ and ${\bar{K}}^{\left(0\right)}=\frac{1}{N}\sum_{i=1}^{N}k_{i}^{\left(0\right)},$ where $k_{i}^{\left(0\right)}=\frac{y_{i}}{x_{i}^{\left(0\right)}}=\frac{y_{i}}{x_{i}}$, we get the usual Hartley-Ross estimator and its variance as:
$${\bar{y}}_{\left(HR\right)}^{\left(U\right)}\approx \bar{y}+{\bar{k}}^{\left(0\right)}\left({\bar{X}}^{\left(0\right)}-{\bar{x}}^{\left(0\right)}\right),$$ (5)
  and
$$Var({\bar{y}}_{\left(HR\right)}^{\left(U\right)})\approx ϒ\left[S_{y}^{2}+{\bar{K}}^{(0)2}S_{x^{\left(0\right)}}^{2}-2{\bar{K}}^{\left(0\right)}S_{yx^{\left(0\right)}}\right].$$ (6)
2. Put *c* = *C*_*x*_, *d* = *B*_2(*x*)_, in (3), so *j* = 1, i.e. ${\bar{x}}^{\left(1\right)}=C_{x}\bar{x}+\beta_{2(x)}$, ${\bar{X}}^{\left(1\right)}=C_{x}\bar{X}+\beta_{2(x)}$, ${\bar{k}}^{\left(1\right)}=\frac{1}{n}\sum_{i=1}^{n}k_{i}^{\left(1\right)},$ and ${\bar{K}}^{\left(1\right)}=\frac{1}{N}\sum_{i=1}^{N}k_{i}^{\left(1\right)}$, where $k_{i}^{\left(1\right)}=\frac{y_{i}}{x_{i}^{\left(1\right)}}=\frac{y_{i}}{C_{x}x_{i}+\beta_{2(x)}}$, we get the [4] estimator with its variance, are given by:
$${\bar{y}}_{\left(S_{1}\right)}^{\left(U\right)}\approx \bar{y}+{\bar{k}}^{\left(1\right)}\left({\bar{X}}^{\left(1\right)}-{\bar{x}}^{\left(1\right)}\right),$$ (7)
  and
$$Var({\bar{y}}_{\left(S_{1}\right)}^{\left(U\right)})\approx ϒ\left[S_{y}^{2}+{\bar{K}}^{(1)2}S_{x^{\left(1\right)}}^{2}-2{\bar{K}}^{\left(1\right)}S_{yx^{\left(1\right)}}\right].$$ (8)
3. Put *c* = *C*_*x*_, *d* = *ρ*_*yx*_, in (3), so *j* = 2, i.e. ${\bar{x}}^{\left(2\right)}=C_{x}\bar{x}+\rho_{yx}$, ${\bar{X}}^{\left(2\right)}=C_{x}\bar{X}+\rho_{yx}$, ${\bar{k}}^{\left(2\right)}=\frac{1}{n}\sum_{i=1}^{n}k_{i}^{\left(2\right)},$ and ${\bar{K}}^{\left(2\right)}=\frac{1}{N}\sum_{i=1}^{N}k_{i}^{\left(2\right)},$ where $k_{i}^{\left(2\right)}=\frac{y_{i}}{x_{i}^{\left(2\right)}}=\frac{y_{i}}{C_{x}x_{i}+\rho_{yx}}$, we get another [4] estimator and its variance, is given by:
$${\bar{y}}_{\left(S2\right)}^{\left(U\right)}\approx \bar{y}+{\bar{k}}^{\left(2\right)}\left({\bar{X}}^{\left(2\right)}-{\bar{x}}^{\left(2\right)}\right),$$ (9)
  and
$$Var({\bar{y}}_{\left(S_{2}\right)}^{\left(U\right)})\approx ϒ\left[S_{y}^{2}+{\bar{K}}^{(2)2}S_{x^{\left(2\right)}}^{2}-2{\bar{K}}^{\left(2\right)}S_{yx^{\left(2\right)}}\right].$$ (10)
4. A difference type estimator using a single auxiliary variable with its ranks, is given by:
$${\bar{y}}_{\left(D\right)}^{\left(U\right)}=\left[\bar{y}+d_{1}(\bar{X}-\bar{x})+d_{2}(\bar{R}-\bar{r})\right],$$ (11)
  where *d*_*i*_(*i* = 1, 2) are constants.
  The variance of ${\bar{y}}_{\left(D\right)}^{\left(U\right)}$, is given by
$$Var({\bar{y}}_{\left(D\right)}^{\left(U\right)})=\left\{\gamma {\bar{Y}}^{2}C_{y}^{2}+d_{1}^{2}{\bar{X}}^{2}m_{1}+d_{2}^{2}{\bar{R}}^{2}m_{2}-2d_{1}\bar{Y}\bar{X}m_{3}-2d_{2}\bar{Y}\bar{R}m_{4}+2d_{1}d_{2}\bar{X}\bar{R}m_{5}\right\},$$
  where
$$m_{1}=ϒC_{x}^{2},m_{2}=ϒC_{r}^{2},m_{3}=ϒC_{yx},m_{4}=ϒC_{yr},m_{5}=ϒC_{xr}.$$
  The minimum variance of ${\bar{y}}_{\left(D\right)}^{\left(U\right)}$, at optimum values of *d*_*i*_(*i* = 1, 2) i.e. $d_{1(opt)}=\frac{\bar{Y}}{\bar{X}}\left(\frac{m_{2}m_{3}-m_{4}m_{5}}{m_{1}m_{2}-m_{5}^{2}}\right)$ and $d_{2(opt)}=\frac{\bar{Y}}{\bar{R}}\left(\frac{m_{1}m_{4}-m_{3}m_{5}}{m_{1}m_{2}-m_{5}^{2}}\right)$, is given by
$$Var{\left({\bar{y}}_{\left(D\right)}^{\left(U\right)}\right)}_{\text{min}}\approx {\bar{Y}}^{2}\left[ϒC_{y}^{2}-\frac{\Delta_{1}}{\left(m_{1}m_{2}-m_{5}^{2}\right)}\right],$$ (12)
  where
$$\Delta_{1}=m_{1}m_{4}^{2}+m_{2}m_{3}^{2}-2m_{3}m_{4}m_{5}.$$
5. [6] suggested the following unbiased estimator using the single auxiliary variable and is given by:
$${\bar{y}}_{\left(CK\right)}^{\left(U\right)}=Q\bar{y}{\left(\frac{c\bar{X}+d}{\alpha \left(c_{}\bar{x}+d\right)+\left(1-\alpha \right)\left(c_{}\bar{X}+d\right)}\right)}^{t}-\left(Q-1\right)\bar{y}-Qϒ\bar{y}\left[\frac{t(t+1)}{2}{ϒ}^{2}\alpha^{2}C_{x}^{2}-\alpha ϒt\frac{s_{yx}}{\bar{y}\bar{X}}\right],$$
  where *c* and *d* are defined earlier i.e. t = −1, 0, 1; *α* = 0, 1 and *Q* is a constant whose value is to be estimated.
  For *α* = *t* = 1, the above estimator becomes:
$${\bar{y}}_{\left(CK\right)}^{\left(U\right)}=Q\bar{y}\left(\frac{c\bar{X}+d}{c_{}\bar{x}+d}\right)-\left(Q-1\right)\bar{y}-Qϒ\bar{y}\left[g^{2}C_{x}^{2}-g\frac{s_{yx}}{\bar{y}\bar{X}}\right],$$ (13)
  $g=\frac{c\bar{X}}{c\bar{X}+d}$.
  The minimum variance of ${\bar{y}}_{\left(CK\right)}^{\left(U\right)}$ at optimum values of *Q* i.e. $Q_{opt}=-\frac{\nabla_{2}}{\nabla_{1}}$, is given by
$$Var{\left({\bar{y}}_{\left(CK\right)}^{\left(U\right)}\right)}_{\text{min}}\approx {\bar{Y}}^{2}\left[ϒC_{y}^{2}-\frac{\nabla_{2}^{2}}{\nabla_{1}}\right],$$ (14)
  where ∇_1_ = ∇_1*a*_ + ∇_1*b*_,
$$\nabla_{1a}={ϒ}^{3}g^{4}C_{x}^{4}C_{y}^{2}+ϒg^{2}C_{x}^{2}+{ϒ}^{2}C_{yx}gC_{y}\frac{\Delta_{210}}{\rho_{yx}}-{ϒ}^{2}g^{4}C_{x}^{4}-{ϒ}^{2}g^{2}C_{yx}^{2}+{ϒ}^{3}C_{yx}^{2}g^{2}\left(\frac{\Delta_{220}}{\rho_{yx}}-1\right),$$
$$\nabla_{1b}=4{ϒ}^{2}g^{3}C_{yx}C_{x}^{2}-2{ϒ}^{2}g^{2}C_{yx}C_{x}\frac{\Delta_{120}}{\rho_{yx}}-2{ϒ}^{3}g^{3}C_{yx}C_{x}^{2}C_{y}\frac{\Delta_{210}}{\rho_{yx}},$$
$$\nabla_{2}=-ϒgC_{yx}-{ϒ}^{2}g^{2}C_{x}^{2}C_{y}^{2}+{ϒ}^{2}gC_{yx}C_{y}\frac{\Delta_{210}}{\rho_{yx}}.$$
6. [8] suggested an idea of using rank of the auxiliary variable in the following estimator, is given by
$${\bar{y}}_{\left(H\right)}=\left[H_{1}\bar{y}+H_{2}(\bar{X}-\bar{x})+H_{3}(\bar{R}-\bar{r})\right]\text{exp}\left(\frac{c(\bar{X}-\bar{x})}{c(\bar{X}+\bar{x})+2d}\right),$$ (15)
  where *H*_*i*_(*i* = 1,2,3) are constants; *c* and *d* are defined earlier.
  The bias of the estimator ${\bar{y}}_{\left(H\right)}$, is given by
$$B({\bar{y}}_{\left(H\right)})\approx (H_{1}-1)\bar{Y}+H_{1}\bar{Y}ϒ\left(\frac{3}{8}g^{2}C_{x}^{2}-\frac{1}{2}gC_{yx}\right)+H_{2}\bar{X}\frac{1}{2}gϒC_{x}^{2}+H_{3}\bar{R}\frac{1}{2}gϒC_{xr}.$$ (16)

Since $\bar{X}$ and $\bar{R}$ are known, so replacing $\bar{Y}$ and *C*_*yx*_ by their consistent estimates $\bar{y}$ and ${\hat{C}}_{yx}=\frac{s_{yx}}{\bar{y}\bar{X}}$ in (16), the estimated bias of ${\bar{y}}_{\left(H\right)}$ becomes

$$\hat{B}({\bar{y}}_{\left(H\right)})\approx (H_{1}-1)\bar{y}+H_{1}\bar{y}ϒ\left(\frac{3}{8}g^{2}C_{x}^{2}-\frac{1}{2}g\frac{s_{yx}}{\bar{y}\bar{X}}\right)+H_{2}\bar{X}\frac{1}{2}gϒC_{x}^{2}+H_{3}\bar{R}\frac{1}{2}gϒC_{xr}.$$ (17)

Subtracting $\hat{B}({\bar{y}}_{\left(H\right)})$ from ${\bar{y}}_{\left(H\right)}$, we get an unbiased estimator by replacing *H*_*i*_(*i* = 1,2,3) by *L*_*i*_(*i* = 1,2,3) which is considered by [10] as:

$${\bar{y}}_{\left(I\right)}^{\left(U\right)}={\bar{y}}_{\left(H\right)}-\hat{B}({\bar{y}}_{\left(H\right)})$$ (18)

or

$$\begin{array}{cc} {\bar{y}}_{\left(I\right)}^{\left(U\right)}= & \left[L_{1}\bar{y}+L_{2}(\bar{X}-\bar{x})+L_{3}(\bar{R}-\bar{r})\right]\text{exp}\left(\frac{c(\bar{X}-\bar{x})}{c(\bar{X}+\bar{x})+2d}\right)-(L_{1}-1)\bar{y}\\ & -L_{1}\bar{y}ϒ\left(\frac{3}{8}g^{2}C_{x}^{2}-\frac{1}{2}g\frac{s_{yx}}{\bar{y}\bar{X}}\right)-L_{2}\bar{X}\frac{1}{2}gϒC_{x}^{2}-L_{3}\bar{R}\frac{1}{2}gϒC_{xr}. \end{array}$$ (19)

Rewriting in terms of errors, we have

$$\begin{array}{cc} {\bar{y}}_{\left(I\right)}^{\left(U\right)}-\bar{Y} & \approx \bar{Y}\Psi_{0}+L_{1}\bar{Y}\left[-\frac{3}{8}ϒg^{2}C_{x}^{2}\Psi_{0}-\frac{1}{2}g\Psi_{1}+\frac{1}{2}ϒC_{yx}g\Psi_{3}-\frac{1}{2}g\Psi_{0}\Psi_{1}+\frac{3}{8}g^{2}\Psi_{1}^{2}\right.\\ & \left.-\frac{3}{8}g^{2}ϒC_{x}^{2}+\frac{1}{2}ϒgC_{yx}\right]-L_{2}\bar{X}\left[\Psi_{1}-\frac{1}{2}g\Psi_{1}^{2}+\frac{1}{2}gϒC_{x}^{2}\right]\\ & -L_{3}\bar{R}\left[\Psi_{2}-\frac{1}{2}g\Psi_{1}\Psi_{2}+\frac{1}{2}gϒC_{xr}\right] \end{array}$$ (20)

Solving (20), the bias of ${\bar{y}}_{\left(I\right)}^{\left(U\right)}$ becomes zero. Now squaring and taking expectation of (20), the variance of ${\bar{y}}_{\left(I\right)}^{\left(U\right)}$ becomes:

$$\begin{array}{cc} Var({\bar{y}}_{\left(I\right)}^{\left(U\right)})= & ϒ{\bar{Y}}^{2}C_{y}^{2}+L_{1}^{2}{\bar{Y}}^{2}h_{1}+L_{2}^{2}{\bar{X}}^{2}h_{2}+L_{3}^{2}{\bar{R}}^{2}h_{3}+2L_{1}{\bar{Y}}^{2}h_{4}-2L_{2}{\bar{Y}}_{}\bar{X}h_{5}\\ & -2L_{3}{\bar{Y}}_{}\bar{R}h_{6}-2L_{1}L_{2}{\bar{Y}}_{}\bar{X}h_{7}-2L_{1}L_{3}{\bar{Y}}_{}\bar{R}h_{8}+2L_{2}L_{3}{\bar{X}}_{}\bar{R}h_{9}, \end{array}$$ (21)

where

$$\begin{array}{cc} h_{1}= & \frac{1}{4}ϒg^{2}C_{x}^{2}-\frac{9}{64}{ϒ}^{2}g^{4}C_{x}^{4}-\frac{1}{4}{ϒ}^{2}g^{2}C_{yx}^{2}-\frac{1}{2}{ϒ}^{2}g^{2}C_{yx}C_{x}\frac{\Delta_{120}}{\rho_{yx}}+\frac{3}{4}{ϒ}^{2}g^{3}C_{yx}C_{x}^{2}\\ & +\frac{9}{64}{ϒ}^{3}g^{4}C_{y}^{2}C_{x}^{4}+\frac{1}{4}{ϒ}^{3}g^{2}C_{yx}^{2}\left(\frac{\Delta_{220}}{\rho_{yx}}-1\right)-\frac{3}{8}{ϒ}^{3}g^{3}C_{x}^{2}C_{yx}C_{y}\left(\frac{\Delta_{210}}{\rho_{yx}}\right), \end{array}$$

$$h_{2}=ϒC_{x}^{2}-\frac{1}{4}{ϒ}^{2}g^{2}C_{x}^{4},$$

$$h_{3}=ϒC_{r}^{2}-\frac{1}{4}{ϒ}^{2}g^{2}C_{xr}^{2},$$

$$h_{4}=-\frac{3}{8}{ϒ}^{2}g^{2}C_{y}^{2}C_{x}^{2}+\frac{1}{2}{ϒ}^{2}gC_{yx}C_{y}\frac{\Delta_{210}}{\rho_{yx}}-\frac{1}{2}ϒgC_{yx},$$

$$h_{5}=ϒC_{yx},$$

$$h_{6}=ϒC_{yr},$$

$$h_{7}=-\frac{5}{8}{ϒ}^{2}g^{2}C_{yx}C_{x}^{2}-\frac{1}{2}ϒgC_{x}^{2}+\frac{1}{2}{ϒ}^{2}gC_{yx}C_{x}\frac{\Delta_{120}}{\rho_{yx}}+\frac{3}{16}{ϒ}^{2}g^{3}C_{x}^{4},$$

$$h_{8}=-\frac{3}{8}{ϒ}^{2}g^{2}C_{x}^{2}C_{yr}-\frac{1}{2}ϒgC_{xr}+\frac{1}{2}{ϒ}^{2}gC_{yx}C_{r}\frac{\Delta_{102}}{\rho_{yr}}+\frac{3}{16}{ϒ}^{2}g^{3}C_{x}^{2}C_{rz}-\frac{1}{4}{ϒ}^{2}g^{2}C_{yx}C_{xr},$$

$$h_{9}=ϒC_{xr}-\frac{1}{4}{ϒ}^{2}g^{2}C_{x}^{2}C_{xr}.$$

The optimum values *L*_*i*_(*i* = 1,2,3) are $L_{1(opt)}=-\frac{{Τ}_{2}}{{Τ}_{1}}$, $L_{2(opt)}=\frac{\bar{Y}}{\bar{X}}\left(\frac{{Τ}_{3}}{{Τ}_{1}}\right)$, $L_{3(opt)}=\frac{\bar{Y}}{\bar{R}}\left(\frac{{Τ}_{4}}{{Τ}_{1}}\right)$.

The minimum variance of ${\bar{y}}_{\left(I\right)}^{\left(U\right)}$ is given by

$$Var{\left({\bar{y}}_{\left(I\right)}^{\left(U\right)}\right)}_{\text{min}}\approx {\bar{Y}}^{2}\left[ϒC_{y}^{2}-\frac{{Τ}_{5}}{{Τ}_{1}}\right],$$ (22)

where

$${\text{T}}_{1}=h_{1}h_{2}h_{3}-h_{1}h_{9}^{2}-h_{2}h_{8}^{2}-h_{3}h_{7}^{2}+2h_{7}h_{8}h_{9},$$

$${\text{T}}_{2}=h_{2}h_{3}h_{4}-h_{2}h_{6}h_{8}-h_{3}h_{5}h_{7}-h_{4}h_{9}^{2}+h_{5}h_{8}h_{9}+h_{6}h_{7}h_{8},$$

$${\text{T}}_{3}=h_{1}h_{3}h_{5}-h_{1}h_{6}h_{9}-h_{3}h_{4}h_{7}+h_{4}h_{8}h_{9}-h_{5}h_{8}^{2}+h_{6}h_{7}h_{8},$$

$${\text{T}}_{4}=h_{1}h_{2}h_{6}-h_{1}h_{5}h_{9}-h_{2}h_{4}h_{8}+h_{4}h_{7}h_{9}+h_{5}h_{7}h_{8}-h_{6}h_{7}^{2},$$

$${\text{T}}_{5}={\text{T}}_{5a}+{\text{T}}_{5b},$$

$${\text{T}}_{5a}=h_{1}h_{2}h_{6}^{2}+h_{2}h_{3}h_{4}^{2}-2h_{2}h_{4}h_{6}h_{8}+h_{1}h_{3}h_{5}^{2}-2h_{1}h_{5}h_{6}h_{9}-2h_{3}h_{4}h_{5}h_{7},$$

$${\text{T}}_{5b}=-h_{4}^{2}h_{9}^{2}+2h_{4}h_{5}h_{8}h_{9}+2h_{4}h_{6}h_{7}h_{9}-h_{5}^{2}h_{8}^{2}+2h_{5}h_{6}h_{7}h_{8}-h_{6}^{2}h_{7}^{2}.$$

## 4. Proposed almost unbiased estimator

On the lines of [1, 8, 10], we propose the following alternative new unbiased estimator. This estimator is based on usual ratio, difference, and exponential ratio type estimators. The purpose is to construct an unbiased estimator that should be better than all considered estimators in estimating the finite population mean.

$${\bar{y}}_{\left(P\right)}=\left[S_{1}\bar{y}\left(\frac{c\bar{X}+d}{c_{}\bar{x}+d}\right)+S_{2}(\bar{X}-\bar{x})+S_{3}(\bar{R}-\bar{r})\right]\text{exp}\left(\frac{c\left(\bar{X}-\bar{x}\right)}{c\left(\bar{X}+\bar{x}\right)+2d}\right),$$ (23)

where *S*_*i*_(*i* = 1,2,3) are constants.

Rewriting (23) in terms of errors, we have

$$\begin{array}{cc} {\bar{y}}_{\left(P\right)}-\bar{Y}= & \left(S_{1}-1\right)\bar{Y}+S_{1}\bar{Y}\left[\Psi_{0}-\frac{3}{2}g\Psi_{1}-\frac{3}{2}g\Psi_{0}\Psi_{1}+\frac{15}{8}g^{2}\Psi_{1}^{2}\right]\\ & -S_{2}\bar{X}\left[\Psi_{1}-\frac{1}{2}g\Psi_{1}^{2}\right]-S_{3}\bar{R}\left[\Psi_{2}-\frac{1}{2}g\Psi_{1}\Psi_{2}\right] \end{array}$$ (24)

From (24), the bias of ${\bar{y}}_{\left(P\right)}$, is given by

$$B({\bar{y}}_{\left(P\right)})\approx (S_{1}-1)\bar{Y}+S_{1}\bar{Y}ϒ\left[\frac{15}{8}g^{2}C_{x}^{2}-\frac{3}{2}gC_{yx}\right]+S_{2}\frac{1}{2}\bar{X}gϒC_{x}^{2}+S_{3}\frac{1}{2}\bar{R}gϒC_{xr}.$$ (25)

The estimated bias of ${\bar{y}}_{\left(P\right)}$, is given by

$$\hat{B}({\bar{y}}_{\left(P\right)})\approx (S_{1}-1)\bar{y}+S_{1}\bar{y}ϒ\left[\frac{15}{8}g^{2}C_{x}^{2}-\frac{3}{2}g\frac{s_{yx}}{\bar{y}\bar{X}}\right]+S_{2}\frac{1}{2}\bar{X}gϒC_{x}^{2}+S_{3}\frac{1}{2}\bar{R}g\Psi C_{xr}.$$ (26)

Subtracting estimated bias given in (26) from the proposed estimator given in (23), the unbiased proposed estimator becomes:

$${\bar{y}}_{\left(P\right)}^{\left(U\right)}={\bar{y}}_{\left(P\right)}-\hat{B}({\bar{y}}_{\left(P\right)})$$ (27)

or

$$\begin{array}{cc} {\bar{y}}_{\left(P\right)}^{\left(U\right)}= & \left[S_{1}\bar{y}\left(\frac{c\bar{X}+d}{c_{}\bar{x}+d}\right)+S_{2}(\bar{X}-\bar{x})+S_{3}(\bar{R}-\bar{r})\right]\text{exp}\left(\frac{c\left(\bar{X}-\bar{x}\right)}{c\left(\bar{X}+\bar{x}\right)+2d}\right)\\ & -(S_{1}-1)\bar{y}-S_{1}\bar{y}ϒ\left[\frac{15}{8}g^{2}C_{x}^{2}-\frac{3}{2}g\frac{s_{yx}}{\bar{y}\bar{X}}\right]-S_{2}\frac{1}{2}\bar{X}gϒC_{x}^{2}-S_{3}\frac{1}{2}\bar{R}gϒC_{xr} \end{array}$$ (28)

Solving (28) in terms of errors, we have

$$\begin{array}{cc} {\bar{y}}_{\left(P\right)}^{\left(U\right)}-\bar{Y}= & \bar{Y}\Psi_{0}+S_{1}\bar{Y}\left[-\frac{15}{8}ϒg^{2}C_{x}^{2}\Psi_{0}-\frac{3}{2}g\Psi_{1}+\frac{3}{2}C_{yx}ϒg\Psi_{3}+\frac{3}{2}ϒgC_{yx}\right.\\ & \left.-\frac{15}{8}ϒg^{2}C_{x}^{2}+\frac{15}{8}g^{2}\Psi_{1}^{2}-\frac{3}{2}g\Psi_{0}\Psi_{1}\right]-S_{2}\bar{X}\left[\Psi_{1}-\frac{1}{2}g\Psi_{1}^{2}+\frac{1}{2}ϒgC_{x}^{2}\right]\\ & -S_{3}\bar{R}\left[\Psi_{2}-\frac{1}{2}g\Psi_{1}\Psi_{2}+\frac{1}{2}gϒC_{xr}\right] \end{array}$$ (29)

From (29), we have

$$B({\bar{y}}_{\left(P\right)}^{\left(U\right)})=0.$$ (30)

Squaring (29) and then taking expectation, we get the variance of ${\bar{y}}_{\left(P\right)}^{\left(U\right)}$ as:

$$\begin{array}{cc} Var({\bar{y}}_{\left(P\right)}^{\left(U\right)})\approx & ϒ{\bar{Y}}^{2}C_{y}^{2}+S_{1}^{2}{\bar{Y}}^{2}q_{1}+S_{2}^{2}{\bar{X}}^{2}q_{2}+S_{3}^{2}{\bar{R}}^{2}q_{3}+2S_{1}{\bar{Y}}^{2}q_{4}-2S_{2}{\bar{Y}}_{}\bar{X}q_{5}\\ & -2S_{3}{\bar{Y}}_{}\bar{Z}q_{6}-2S_{1}S_{2}{\bar{Y}}_{}\bar{X}q_{7}-2S_{1}S_{3}{\bar{Y}}_{}\bar{R}q_{8}+2S_{2}S_{3}\bar{R}\bar{X}q_{9}, \end{array}$$ (31)

where

$$\begin{array}{cc} q_{1}= & \frac{225}{64}g^{4}{ϒ}^{3}C_{x}^{4}C_{y}^{2}+\frac{9}{4}ϒg^{2}C_{x}^{2}+\frac{9}{4}{ϒ}^{3}g^{2}C_{yx}^{2}\left(\frac{\Delta_{220}}{\rho_{yx}}-1\right)-\frac{9}{4}{ϒ}^{2}g^{2}C_{yx}^{2}-\frac{225}{64}{ϒ}^{2}g^{4}C_{x}^{4}\\ & -\frac{45}{8}{ϒ}^{3}g^{3}C_{x}^{2}C_{yx}C_{y}\frac{\Delta_{210}}{\rho_{yx}}-\frac{9}{2}{ϒ}^{2}g^{2}C_{yx}C_{x}\frac{\Delta_{120}}{\rho_{yx}}+\frac{45}{4}{ϒ}^{2}g^{3}C_{yx}C_{x}^{2}, \end{array}$$

$$q_{2}=ϒC_{x}^{2}\left(1-\frac{1}{2}ϒg^{2}C_{x}^{2}\right),$$

$$q_{3}=ϒ\left(C_{r}^{2}-\frac{1}{2}ϒg^{2}C_{xr}^{2}\right),$$

$$q_{4}=-\frac{15}{8}{ϒ}^{2}g^{2}C_{y}^{2}C_{x}^{2}-\frac{3}{2}ϒgC_{yx}+\frac{3}{2}{ϒ}^{2}gC_{yx}C_{y}\frac{\Delta_{210}}{\rho_{yx}},$$

$$q_{5}=ϒC_{yx},$$

$$q_{6}=ϒC_{yr},$$

$$q_{7}=-\frac{15}{8}{ϒ}^{2}g^{2}C_{yx}C_{x}^{2}-\frac{3}{2}ϒgC_{x}^{2}+\frac{3}{2}{ϒ}^{2}gC_{yx}C_{x}\frac{\Delta_{120}}{\rho_{yx}}+\frac{1}{2}{ϒ}^{2}g^{3}C_{x}^{4},$$

$$q_{8}=-\frac{15}{8}{ϒ}^{2}g^{2}C_{x}^{2}C_{yr}-\frac{3}{2}ϒgC_{xr}+\frac{3}{2}{ϒ}^{2}gC_{yx}C_{r}\frac{\Delta_{102}}{\rho_{yr}}+\frac{15}{16}{ϒ}^{2}g^{3}C_{x}^{2}C_{xr}-\frac{3}{4}{ϒ}^{2}g^{2}C_{yx}C_{xr},$$

$$q_{9}=ϒC_{xr}-\frac{1}{4}{ϒ}^{2}g^{2}C_{x}^{2}C_{xr}.$$

The optimum values of *S*_*i*_(*i* = 1,2,3) are $S_{1(opt)}=-\frac{{℧}_{2}}{{℧}_{1}}$, $S_{2(opt)}=\frac{\bar{Y}}{\bar{X}}\left(\frac{{℧}_{3}}{{℧}_{1}}\right)$, $S_{3(opt)}=\frac{\bar{Y}}{\bar{R}}\left(\frac{{℧}_{4}}{{℧}_{1}}\right)$, where

$${℧}_{1}=q_{1}q_{2}q_{3}-q_{1}q_{9}^{2}-q_{2}q_{8}^{2}-q_{3}q_{7}^{2}+2q_{7}q_{8}q_{9},$$

$${℧}_{2}=q_{2}q_{3}q_{4}-q_{2}q_{6}q_{8}-q_{3}q_{5}q_{7}-q_{4}q_{9}^{2}+q_{5}q_{8}q_{9}+q_{6}q_{7}q_{9},$$

$${℧}_{3}=q_{1}q_{3}q_{5}-q_{1}q_{6}q_{9}-q_{3}q_{4}q_{7}+q_{4}q_{8}q_{9}-q_{5}q_{8}^{2}+q_{6}q_{7}q_{8},$$

$${℧}_{4}=q_{1}q_{2}q_{6}-q_{1}q_{5}q_{9}-q_{2}q_{4}q_{8}+q_{4}q_{7}q_{9}+q_{5}q_{7}q_{8}-q_{6}q_{7}^{2}.$$

Substituting the optimum values of *S*_*i*_(*i* = 1,2,3) in (31), we get the minimum variance of ${\bar{y}}_{\left(P\right)}^{\left(U\right)}$, which is given by

$$Var{\left({\bar{y}}_{\left(P\right)}^{\left(U\right)}\right)}_{\text{min}}\approx {\bar{Y}}^{2}\left[ϒC_{y}^{2}-\frac{{℧}_{5}}{{℧}_{1}}\right],$$ (32)

where

$${℧}_{5}={℧}_{5a}+{℧}_{5b},$$

$${℧}_{5a}=q_{1}q_{2}q_{6}^{2}+q_{2}q_{3}q_{4}^{2}-2q_{2}q_{5}q_{6}q_{8}+q_{1}q_{3}q_{5}^{2}-2q_{1}q_{5}q_{6}q_{9}-2q_{3}q_{4}q_{5}q_{7},$$

$${℧}_{5b}=-q_{4}^{2}q_{9}^{2}+2q_{4}q_{5}q_{8}q_{9}+2q_{4}q_{6}q_{7}q_{9}-q_{5}^{2}q_{8}^{2}+2q_{5}q_{6}q_{7}q_{8}-q_{6}^{2}q_{7}^{2}.$$

## 5. Numerical example

We use the following 4 real data sets for a numerical study.

**Population 1**: [source: [13]]
  *Y* = Number of tube wells, *X* = Net irrigated area.
  *N* = 69, *n* = 10, $\bar{Y}=135.2609$, $\bar{X}=345.7536$, $\bar{R}=34.9565$, ${\bar{K}}^{\left(0\right)}=0.4246$, ${\bar{K}}^{\left(1\right)}=0.4981$, ${\bar{K}}^{\left(2\right)}=0.4804$, *C*_*y*_ = 0.8421, *C*_*x*_ = 0.8478, *C*_*r*_ = 0.5731, *ρ*_*yx*_ = 0.9224, *ρ*_*yr*_ = 0.7140, *ρ*_*xr*_ = 0.8193, Δ_220_ = 8.0922, Δ_210_ = 2.1398, Δ_120_ = 2.1183, Δ_102_ = 0.3920, *β*_1*x*_ = 2.3808, *β*_2*x*_ = 7.2159.
**Population 2**: [source: [13]]
  *Y* = Number of tube wells, *X* = Number of tractors.
  *N* = 69, *n* = 10, $\bar{Y}=135.2609$, $\bar{X}=21.2319$, $\bar{R}=34.1159$, ${\bar{K}}^{\left(0\right)}=7.1821$, ${\bar{K}}^{\left(1\right)}=8.0432$, ${\bar{K}}^{\left(2\right)}=6.3598$, *C*_*y*_ = 0.8421, *C*_*x*_ = 0.7969, *C*_*r*_ = 0.8654, *ρ*_*yx*_ = 0.9118, *ρ*_*yr*_ = 0.7409, *ρ*_*xr*_ = 0.8654, Δ_220_ = 6.7066, Δ_210_ = 1.9555, Δ_120_ = 1.8119, Δ_102_ = 0.3825, *β*_1*x*_ = 1.855, *β*_2*x*_ = 3.7632.
**Population 3**: [source: [14]]
  This data set is based on Marmara region of Turkey in 2007.
  *Y* = Number of teachers, *X* = Number of classes.
  *N* = 127, *n* = 31, $\bar{Y}=703.74$, $\bar{X}=498.28$, $\bar{R}=63.8897$, ${\bar{K}}^{\left(0\right)}=1.2071$, ${\bar{K}}^{\left(1\right)}=0.8906$, ${\bar{K}}^{\left(2\right)}=1.0663$, *C*_*y*_ = 1.2559, *C*_*x*_ = 1.1150, *C*_*r*_ = 0.5769, *ρ*_*yx*_ = 0.9789, *ρ*_*yr*_ = 0.8312, *ρ*_*xr*_ = 0.8516, Δ_220_ = 4.7079, Δ_210_ = 1.6136, Δ_120_ = 1.6171, Δ_102_ = 0.4233, *β*_1*x*_ = 1.7205, *β*_2*x*_ = 2.3149.
**Population 4**: [source: [14]]
  This data set is based on Marmara region of Turkey in 2007.
  *Y* = Number of teachers, *X* = Number of students.
  *N* = 127, *n* = 31, $\bar{Y}=703.74$, $\bar{X}=20804.59$, $\bar{R}=64.0$, ${\bar{K}}^{\left(0\right)}=0.0433$, ${\bar{K}}^{\left(1\right)}=0.0296$, ${\bar{K}}^{\left(2\right)}=0.0295$, *C*_*y*_ = 1.2559, *C*_*x*_ = 1.4654, *C*_*r*_ = 0.5751, *ρ*_*yx*_ = 0.9366, *ρ*_*yr*_ = 0.8240, *ρ*_*xr*_ = 0.7834, Δ_220_ = 4.7079, Δ_210_ = 1.5674, Δ_120_ = 1.7115, Δ_102_ = 0.4015, *β*_1*x*_ = 2.1638, *β*_2*x*_ = 4.5928.

The results based on Populations 1–4 are given in Tables 1–4. Tables 1–4 give the results when no conventional measures and conventional measures are used. We use the following expression to obtain the percent relative efficiency (PRE) as:

$$\text{PRE}=\frac{Var({\bar{y}}_{\left(0\right)}^{\left(U\right)})}{Var({\bar{y}}_{\left(i\right)}^{\left(U\right)})}\times 100,$$

where *i* = 0, *HR*, *S*_1_, *S*_2_, *D*, *CK*, *I*, *P*.

**Table 1:** Results of different estimators for Population 1.

Estimator	PRE	Parameters		PRE values of three estimators		
		*c*	*d*	${\bar{y}}_{\left(CK\right)}^{\left(U\right)}$	${\bar{y}}_{\left(I\right)}^{\left(U\right)}$	${\bar{y}}_{\left(P\right)}^{\left(U\right)}$
** ${\bar{y}}_{\left(0\right)}^{\left(u\right)}$ **	100.00	1	*C*_*x*_	335.54	738.08	858.78
** ${\bar{y}}_{\left(HR\right)}^{\left(U\right)}$ **	561.22	1	*β*_1*x*_	334.81	737.35	856.32
** ${\bar{y}}_{\left(S_{1}\right)}^{\left(U\right)}$ **	359.21	1	*β*_2*x*_	332.55	735.15	848.92
** ${\bar{y}}_{\left(S_{2}\right)}^{\left(U\right)}$ **	403.69	1	*ρ*_*yx*_	335.51	738.04	858.66
** ${\bar{y}}_{\left(D\right)}^{\left(U\right)}$ **	695.01	*C*_*x*_	1	335.39	737.92	858.24
		*C*_*x*_	*β*_1*x*_	334.61	737.15	855.64
		*C*_*x*_	*β*_2*x*_	331.95	734.58	847.04
		*C*_*x*_	*ρ*_*yx*_	335.43	737.96	858.39
		*β*_1*x*_	1	335.75	738.28	859.47
		*β*_1*x*_	*C*_*x*_	335.78	738.31	859.58
		*β*_1*x*_	*β*_2*x*_	334.50	737.05	855.29
		*β*_1*x*_	*ρ*_*yx*_	335.77	738.29	859.53
		*β*_2*x*_	1	335.90	738.41	859.93
		*β*_2*x*_	*C*_*x*_	335.90	738.42	859.97
		*β*_2*x*_	*β*_1*x*_	335.79	738.32	859.62
		*β*_2*x*_	*ρ*_*yx*_	335.89	738.42	859.95
		*ρ*_*yx*_	1	335.43	737.96	858.39
		*ρ*_*yx*_	*C*_*x*_	335.51	738.04	858.66
		*ρ*_*yx*_	*β*_1*x*_	334.72	737.26	856.00
		*ρ*_*yx*_	*β*_2*x*_	332.27	734.88	848.03

**Table 2:** Results of different estimators for Population 2.

Estimator	PRE	Parameters		PRE values of three estimators		
		*c*	*d*	${\bar{y}}_{\left(CK\right)}^{\left(U\right)}$	${\bar{y}}_{\left(I\right)}^{\left(U\right)}$	${\bar{y}}_{\left(P\right)}^{\left(U\right)}$
${\bar{y}}_{\left(0\right)}^{\left(u\right)}$	100.00	1	*C*_*x*_	309.18	641.20	752.07
${\bar{y}}_{\left(HR\right)}^{\left(U\right)}$	519.04	1	*β*_1*x*_	303.34	636.46	738.04
${\bar{y}}_{\left(S_{1}\right)}^{\left(U\right)}$	402.10	1	*β*_2*x*_	293.93	629.77	718.67
${\bar{y}}_{\left(S_{2}\right)}^{\left(U\right)}$	589.26	1	*ρ*_*yx*_	308.52	640.64	750.39
${\bar{y}}_{\left(D\right)}^{\left(U\right)}$	627.44	*C*_*x*_	1	306.59	639.04	745.63
		*C*_*x*_	*β*_1*x*_	300.89	634.60	732.62
		*C*_*x*_	*β*_2*x*_	289.65	627.10	711.00
		*C*_*x*_	*ρ*_*yx*_	307.20	639.55	747.13
		*β*_1*x*_	1	310.68	642.50	755.94
		*β*_1*x*_	*C*_*x*_	311.33	643.07	757.65
		*β*_1*x*_	*β*_2*x*_	302.43	635.76	735.99
		*β*_1*x*_	*ρ*_*yx*_	310.96	642.75	756.68
		*β*_2*x*_	1	312.31	643.95	760.29
		*β*_2*x*_	*C*_*x*_	312.64	644.24	761.18
		*β*_2*x*_	*β*_1*x*_	310.95	642.74	756.66
		*β*_2*x*_	*ρ*_*yx*_	312.45	644.07	760.68
		*ρ*_*yx*_	1	307.47	639.77	747.79
		*ρ*_*yx*_	*C*_*x*_	308.73	640.83	750.94
		*ρ*_*yx*_	*β*_1*x*_	302.40	635.74	735.93
		*ρ*_*yx*_	*β*_2*x*_	292.28	628.71	715.62

**Table 3:** Results of different estimators for Population 3.

Estimator	PRE	Parameters		PRE values of three estimators		
		*c*	*d*	${\bar{y}}_{\left(CK\right)}^{\left(U\right)}$	${\bar{y}}_{(I)c}^{\left(U\right)}$	${\bar{y}}_{\left(P\right)}^{\left(U\right)}$
${\bar{y}}_{\left(0\right)}^{\left(u\right)}$	100.00	1	*C*_*x*_	1068.26	2449.65	4339.58
${\bar{y}}_{\left(HR\right)}^{\left(U\right)}$	1108.83	1	*β*_1*x*_	1067.57	2449.38	4328.87
${\bar{y}}_{\left(S_{1}\right)}^{\left(U\right)}$	460.04	1	*β*_2*x*_	1066.90	2449.12	4318.48
${\bar{y}}_{\left(S_{2}\right)}^{\left(U\right)}$	729.92	1	*ρ*_*yx*_	1068.42	2449.72	4342.01
${\bar{y}}_{\left(D\right)}^{\left(U\right)}$	2397.13	*C*_*x*_	1	1068.51	2449.75	4343.48
		*C*_*x*_	*β*_1*x*_	1067.77	2449.46	4332.00
		*C*_*x*_	*β*_2*x*_	1067.17	2449.22	4322.64
		*C*_*x*_	*ρ*_*yx*_	1068.53	2449.76	4343.82
		*β*_1*x*_	1	1068.87	2449.89	4349.14
		*β*_1*x*_	*C*_*x*_	1068.79	2449.87	4347.94
		*β*_1*x*_	*β*_2*x*_	1068.00	2449.55	4335.49
		*β*_1*x*_	*ρ*_*yx*_	1068.88	2449.90	4349.37
		*β*_2*x*_	1	1069.04	2449.96	4351.84
		*β*_2*x*_	*C*_*x*_	1068.98	2449.94	4350.94
		*β*_2*x*_	*β*_1*x*_	1068.68	2449.82	4346.23
		*β*_2*x*_	*ρ*_*yx*_	1069.05	2449.97	4352.08
		*ρ*_*yx*_	1	1068.37	2449.70	4341.23
		*ρ*_*yx*_	*C*_*x*_	1068.23	2449.64	4339.16
		*ρ*_*yx*_	*β*_1*x*_	1067.53	2449.37	4328.22
		*ρ*_*yx*_	*β*_2*x*_	1066.84	2449.10	4317.62

**Table 4:** Results of different estimators for Population 4.

Estimator	PRE	Parameters		PRE values of three estimators		
		*c*	*d*	${\bar{y}}_{\left(CK\right)}^{\left(U\right)}$	${\bar{y}}_{\left(I\right)}^{\left(U\right)}$	${\bar{y}}_{\left(P\right)}^{\left(U\right)}$
${\bar{y}}_{\left(0\right)}^{\left(u\right)}$	100.00	1	*C*_*x*_	688.84	964.64	1040.86
${\bar{y}}_{\left(HR\right)}^{\left(U\right)}$	230.94	1	*β*_1*x*_	688.83	964.64	1040.78
${\bar{y}}_{\left(S_{1}\right)}^{\left(U\right)}$	769.77	1	*β*_2*x*_	688.80	964.64	1040.49
${\bar{y}}_{\left(S_{2}\right)}^{\left(U\right)}$	773.16	1	*ρ*_*yx*_	688.85	964.64	1040.92
${\bar{y}}_{\left(D\right)}^{\left(U\right)}$	983.47	*C*_*x*_	1	688.85	964.64	1040.95
		*C*_*x*_	*β*_1*x*_	688.84	964.64	1040.86
		*C*_*x*_	*β*_2*x*_	688.82	964.64	1040.66
		*C*_*x*_	*ρ*_*yx*_	688.85	964.64	1040.96
		*β*_1*x*_	1	688.86	964.64	1040.78
		*β*_1*x*_	*C*_*x*_	688.83	964.64	1040.98
		*β*_1*x*_	*β*_2*x*_	688.86	964.64	1040.78
		*β*_1*x*_	*ρ*_*yx*_	688.86	964.64	1041.01
		*β*_2*x*_	1	688.86	964.64	1040.99
		*β*_2*x*_	*C*_*x*_	688.86	964.64	1040.98
		*β*_2*x*_	*β*_1*x*_	688.86	964.64	1040.98
		*β*_2*x*_	*ρ*_*yx*_	688.86	964.64	1041.01
		*ρ*_*yx*_	1	688.85	964.64	1040.91
		*ρ*_*yx*_	*C*_*x*_	688.84	964.64	1040.85
		*ρ*_*yx*_	*β*_1*x*_	688.83	964.64	1040.76
		*ρ*_*yx*_	*β*_2*x*_	688.79	964.63	1040.46

In Tables 1–4, the proposed unbiased estimator ${\bar{y}}_{\left(P\right)}^{\left(U\right)}$ outperforms in all four Populations but the [4] estimators ${\bar{y}}_{\left(S_{1}\right)}^{\left(U\right)}$ in Populations 1–3 and [1] estimator ${\bar{y}}_{\left(HR\right)}^{\left(U\right)}$ in Population 4 are performing poorly.

## 6. Conclusion

In this study, we have proposed an unbiased class of estimators in estimating the finite population mean in simple random sampling using the single auxiliary variable and rank of the auxiliary variable. Expressions for biases and MSEs or variances are obtained up to first order of approximation. Four data sets are used for numerical study. The proposed estimator outperforms in all four populations as compared to all considered estimators. It is observed that use of conventional measures i.e. *C*_*x*_, *β*_1*x*_, *β*_2*x*_, and *ρ*_*yx*_ have significant role in increasing the efficiency of the estimators in Tables 1–4.

[4] estimators ${\bar{y}}_{\left(S_{1}\right)}^{\left(U\right)}$ in Populations 1–3 and [1] estimator ${\bar{y}}_{\left(HR\right)}^{\left(U\right)}$ in Population 4 show the poor performance but the proposed unbiased estimator ${\bar{y}}_{\left(P\right)}^{\left(U\right)}$ have an excellent performance as compared to all considered estimators in all four populations 1–4.
